# Effects of 1 Gray radiation on the biophysical properties of the tibia and femur from hindlimb‐suspended rats

**DOI:** 10.1113/EP093760

**Published:** 2026-09-19

**Authors:** Brent J. F. Hill, Rahul Mehta

**Affiliations:** ^1^ Department of Biology University of Central Arkansas Conway Arkansas USA; ^2^ Department of Physics, Astronomy, & Engineering University of Central Arkansas Conway Arkansas USA

**Keywords:** 3‐point bending, femur, radiation, stiffness, tibia

## Abstract

Long‐term space travel (e.g. travel to Mars) will greatly exceed the recommended lifetime radiation dose of 600 millisieverts (mSv) and have a detrimental effect on skeletal fragility. Our objective was to determine if 1 Gray (GY) radiation impacts the biomechanical properties of the tibia and femur from rats under microgravity. Hindlimb‐suspended male Sprague–Dawley rats were irradiated (IR) with 1 GY (+IR) or used as control (−IR); after 4 weeks they were sacrificed to obtain the femur and tibia. Using the three‐point bending technique, we found radiation had no effect on the flexure force/bending and stiffness of the femur. However, the tibias from the +IR rats demonstrated 37% less flexure force/bending and stiffness than −IR rats. Bone elemental analysis was determined using a scanning electron microscope equipped with energy dispersive spectroscopy; there was no difference in the calcium/phosphorus ratio between +IR and −IR groups. This study provides pilot data which suggest that low dose radiation exposure has a greater impact on the smaller hindlimb bones from hindlimb‐suspended rats.

## Abstract (highlights)

- **What is the central question of this study?**
  Does low dose radiation (1 Gray) have a detrimental effect on the biomechanical properties of the tibia and femur from rats under microgravity conditions?
- **What is the main finding and its importance?**
  Using hindlimb‐suspended rats, 1 Gray whole body, X‐ray radiation had a negative impact on the biophysical features (flexure force and stiffness) of the tibia, but not the femur. This suggests that bones respond differently to low dose radiation under microgravity conditions that are a ground‐based model for spaceflight.

## 1 INTRODUCTION

In space, microgravity conditions and cosmic radiation have detrimental effects on the skeletal system of humans such as reduced bone resorption, bone mineral density, vascular porosity and capillaries, and lowered elastic moduli (Lekan, 2012; Siamwala et al., 2022). Overall, abnormalities in bone and mineral homeostasis in astronauts during spaceflight results in bone loss (Sibonga, 2013). Biologically, bone has both organic and inorganic components that are interwoven to create a sturdy yet flexible skeletal architecture. The organic components are mainly collagen and long chains of protein, which intertwine as flexible fibres. The inorganic component is hydroxyapatite, a calcium and phosphorus‐rich mineral that strengthens collagen (Sibonga, 2013). In the bone formation process, osteoblasts build the bone while osteoclasts break it down. The calcium/phosphate (Ca/P) ratio can be used to evaluate the osteoblastic activity and health of the bone matrix (Heaney, 2001; Kourkoumelis et al., 2012). One of the best ways to evaluate the mechanical properties of a whole bone at the tissue level is to assess its elastic modulus (Jepsen et al., 2015), defined as a material's resistance to elastic deformation under a force (Currey, 1984). A positive correlation between bone mineral density and elastic modulus has been established at the whole bone level and is commonly used in assessing fracture risk, diagnosing osteoporosis and measuring the efficacy of therapies (Ammann & Rizzoli, 2003; Martin, 1991; Turner, 2002).

Most studies use a higher radiation dose to investigate radiation‐induced changes in bone morphology and strength (Lerouxel et al., 2009; Mendes et al., 2020; Rahman et al., 2018). It has been well documented that the combined effects of radiation and microgravity during spaceflight have a profound effect on bone microarchitecture to induce bone loss (Dong et al., 2021; Gamboa et al., 2021; Keune et al., 2015). However, the combined effects are likely not additive. Some investigators have suggested that microgravity has a greater detrimental impact on bone architecture and strength than radiation (Chowdhury et al., 2016; Ko et al., 2020; Yu et al., 2017). Differences in results are likely due to type and duration of radiation, bone evaluated and duration of hindlimb suspension. For this study we do not evaluate these combined effects, but instead, our aim is to determine if radiation has a detrimental effect on the mechanical features of bones that are under microgravity conditions. Radiation exposure has been shown to damage osteoblast precursors within an irradiated area, but the specific mechanisms and potential influences on bone elasticity are still unknown (Farley et al., 2020; Mehta et al., 2019; NCRP, 1989).

In space, astronauts are exposed to radiation levels many times what is experienced on Earth's surface. Based on the National Aeronautics and Space Administration (NASA) and the National Council on Radiation Protection and Measurements (NCRP) reports (Gamboa et al., 2021; NCRP, 1989, 1993; Polk, 2022), the NASA committee in 2021 revised the spaceflight radiation permissible exposure limit by expressing it as a dose limit of 3% of the mean risk of exposure induced death (REID); an estimate of ∼600 mSv (millisievert) (Polk, 2022; Zeitlin et al., 2013). And, the health hazards will only increase for astronauts during deep and long‐term space travel (e.g. travel to Mars) because their prolonged radiation exposure will exceed 1 Sv (equivalent to 1 GY used in this study) (Yasuda & Sihver, 2022). We anticipate this pilot study will provide information to justify additional experimental studies using low dose radiation to investigate its effect on astronaut health.

## 2 METHODS

### 2.1 Ethical approval

Rats were housed, hindlimb suspended, and irradiated at the Division of Laboratory Animal Medicine at the University of Arkansas for Medical Sciences (UAMS) under the guidance of Dr P. Chowdhury (Professor Emeritus, Department of Physiology & Biophysics, UAMS; see Acknowledgements). The experimental protocol was approved by the UAMS Institutional Animal Care and Use Committee (no. A3063‐01). Because P. Chowdhury did not need the rat hindlimb bones for his study, the authors obtained the dissected rat bones *post mortem* from P. Chowdhury and brought them to the University of Central Arkansas for analysis. Experiments were performed and reported in accordance with the relevant ARRIVE guidelines and regulations.

### 2.2 Animal groups

Two experimental groups of hindlimb‐suspended rats were evaluated: a radiation exposure (+IR, *n* = 4) and non‐radiation (−IR, *n* = 4) control group. Both groups of rats were hindlimb suspended (Chowdhury et al., 2016), housed in the Division of Laboratory Animal Medicine at UAMS, and fed food (Inotiv, Harlan Teklad rodent diet no. 8640, West Lafayette, IN, USA) and water ad libitum. Rats were acclimated for 7 days before undergoing hindlimb suspension and radiation (started on same day). Once experimental interventions began, the rats were individually housed. The radiation group of rats (male Sprague–Dawley rats, 200–225 g, 7 weeks old; Charles River Laboratories, Wilmington, MA, USA) were exposed to 1 GY (Gray, absorbed radiation) of whole body, X‐ray radiation using a Faxitron cabinet X‐ray system (Lincolnshire, IL, USA). Four weeks after being irradiated, rats were initially anaesthetized using an induction chamber and an isoflurane vaporizer set at ∼3%. For euthanasia, isoflurane was increased above 5% and monitored until respiration ceased; exsanguination was used as secondary confirmation. The leg bones (tibia and femur) were dissected out of each rat, wrapped in saline‐soaked gauze and stored at −20°C until experimentation.

### 2.3 Three‐point bending

The bones were subjected to the three‐point bending technique (Deckard et al., 2017; Jepsen et al., 2015), which is used to measure biomechanical parameters such as stress, strain and stiffness. Before experimentation, the bones were soaked in phosphate‐buffered saline. The three‐point bending method fixes bones at both ends while a force probe (flat tipped wedge connected to a motorized force transducer) moves upwards at a rate of 0.498 mm s^−1^ at the centre, and perpendicular to the bones’ surface. We fabricated an acrylic holder that would secure both ends of a bone at a span length of 10 mm. The force probe loaded/bent each bone in the middle of the span length. Force generation was controlled by a Hayden Kerk IDEA drive interface program and recorded by WINDAQ data acquisition software (DATAQ Instruments, Montrose‐Ghent, OH, USA). The force probe was placed at the mid‐diaphysis on the anterior side of each bone and moved upward at a known 3 mm distance and subsequently retracted. Thus, bones were not loaded until failure.

### 2.4 Scanning electron microscopy analysis

Each bone was fixed to a microscope slide using a temporary adhesive (Crystalbond 555; Aremco Products Inc., Valley Cottage, NY, USA). Then, the midpoint of each bone was cross‐sectioned (1–2 mm) using a diamond wafering blade (IsoMet1000 Precision Saw, Buehler, Lake Bluff, IL, USA). Each cross section was rinsed in hot water (to remove the water‐soluble adhesive) and placed on a mounting stub. Bones were sputter coated with gold for analysis using a scanning electron microscope (SEM; JSM‐IT100, JEOL Technics Ltd, Tokyo, Japan). We performed elemental analysis of the bone cross sections using the SEM via energy dispersive spectroscopy (EDS). EDS quantifies the relative percentages of carbon, oxygen, phosphorus, and calcium present in the bone mineral hydroxyapatite. Elemental composition measurements were made on the anterior side using an electron beam of the JEOL SEM (Kourkoumelis et al., 2012). The electron beam incident energy was kept at 15–20 keV, which was sufficiently higher than the ionization energy of K‐ and L‐ shells of the sample bones’ elements that produced the X‐ray. The excited X‐rays (by the electrons) were characteristic of the elements it originated from. The X‐ray data for each element were corrected for the ZAF (Z: atomic number, A: absorption in the layers of the intervening medium before the detector for EDS analysis, F: fluorescence yield of the electron shell). Calcium phosphate samples were used for the SEM EDS calibration and to validate the elemental ratio. Each sample was run for a desired length of time to achieve elemental peak areas (from K‐ and L‐shell X‐rays) that were statistically significant. For Gaussian peaks with Laurential tails, statistical uncertainties (in area measurements) were A ± √A; the uncertainty was kept below 1%.

### 2.5 Cortical and marrow analysis

Each bone cross section was imaged using a Leica MZ6 microscope (Deerfield, IL, USA) and OptixCam digital camera. To calculate the moment of inertia (Deckard et al., 2017), measurements were taken using ImageJ (NIH, Bethesda, MD, USA) of the inner and outer diameters of the medullary cavity and cortical compact bone in the anterior/posterior direction. Measurements of the cortical and marrow area also used this program.

### 2.6 Statistics

Stiffness, moment of inertia and elastic modulus were calculated as previously described (Deckard et al., 2017; Jepsen et al., 2015; Patel et al., 2013; Saffar et al., 2009). Groups (+IR versus −IR) were compared using an unpaired Student's *t*‐test, in which, *P* ≤ 0.05 was considered significant. Data are expressed as the mean ± SD for the number of rats (*n* = 4) used for each group (GraphPad Prism, GraphPad Software, Boston, MA, USA).

## 3 RESULTS

Table 1 summarizes the anatomical dimensions of the tibia and femur in each treatment group. One Gray of whole body, X‐ray radiation did not affect the anatomical dimensions of the tibia and femur between the two treatment groups. The moment of inertia around the anterior–posterior axis of each bone cross‐section can be used to calculate the modulus of elasticity using the Euler–Bernoulli equation (Patel et al., 2013).

**TABLE 1:** Cross sectional properties of the tibia and femur.

Parameter	Tibia			Femur		
	−IR	+IR	*P*	−IR	+IR	*P*
**Cortical area (mm^2^)**	4.10 ± 0.181	3.98 ± 0.331	0.548	5.06 ± 0.304	4.98 ± 0.589	0.796
**Marrow area (mm^2^)**	1.22 ± 0.227	1.38 ± 0.250	0.410	3.73 ± 0.773	3.86 ± 0.800	0.845
**Moment of inertia** **anterior–posterior axis (mm^4^)**	2.76 ± 0.479	2.52 ± 0.471	0.505	4.77 ± 0.688	4.19 ± 0.930	0.352
**Ca/P ratio**	1.97 ± 0.051	1.97 ± 0.052	0.962	1.94 ± 0.028	1.98 ± 0.106	0.549

*Note*: Data are expressed as the mean ± SD. There is no difference between treatment groups (*n* = 4 rats for each group) for each bone. −IR indicates the control, non‐radiation group; +IR indicates the irradiated group.

**FIGURE 1:**
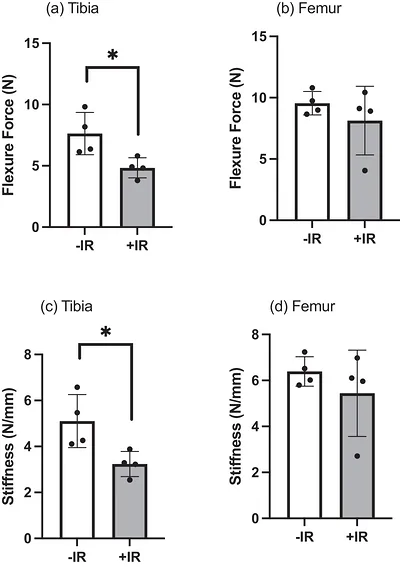
Biophysical properties of the tibia and femur. (a, b) Radiation exposure decreased the mechanical force required to bend (flexure force) the tibia (a; *P* = 0.039) a distance of 3 mm, but not the femur (b; *P* = 0.395). (c, d) Similarly, radiation decreased the stiffness in the tibia (c; *P* = 0.039), but not the femur (d; *P* = 0.395). *Significance, unpaired *t*‐test (*n* = 4 rats). −IR is the control, non‐radiation group; +IR is the irradiated group.

The three‐point bending technique (Jepsen et al., 2015) was used to measure bending/flexure force and stiffness in the tibia and femur. Figure 1 shows the calculated biophysical properties (flexure and stiffness) of the tibia and femur. Radiation appears to significantly decrease both the flexure force on the anterior side of the tibia (*P* = 0.039) and stiffness (*P* = 0.039), both by 37%. There was no impact of radiation on the bending (*P* = 0.395) of the femur or its stiffness (*P* = 0.395). We do not report the tibial elastic modulus because the calculated moment of inertia has some limitations when using the Euler–Bernoulli equation. The equation assumes that the bone possesses a homogeneous cross‐section at the location of loading the bone. The mid‐shaft of the tibia has a triangular orientation, and thus does not have a uniform shape. Because the femur has a more uniform circumferential shape at its cross‐section, we were able to calculate and determine that radiation did not induce any difference (*P* = 0.281) in femur elasticity from the control femur (1065.135 ± 481.191 and 1403.575 ± 281.706 MPa, respectively).

The relative percentages of elements in the bone mineral were determined using energy dispersive spectroscopy. The Ca/P ratio was measured from both +IR and –IR groups. This ratio is a good indicator about the quality of the bone area (Sotiropoulou et al., 2015). The Ca/P ratio was very similar across groups for both the femur and the tibia, thus suggesting that the mineral composition did not change with 1 GY of radiation absorbance (Table 1).

## 4 DISCUSSION

Many studies have evaluated the effect of radiation on bone health. However, there are variations in outcomes depending on type of radiation and duration (Bokhari et al., 2019; Hamilton et al., 2006; Oest et al., 2018). Zeitlin et al., (2013) estimated that adult humans will be exposed to 600–1200 mSv (1 Sv is equal to 1 GY) of cosmic radiation in transit to Mars. Most studies use a higher radiation dose to investigate radiation‐induced changes in bone morphology and strength (Lerouxel et al., 2009; Mendes et al., 2020; Rahman et al., 2018). The aim of this study was to evaluate the biomechanical parameters (flexure force, stiffness and elasticity) of suspended hindlimb bones exposed to an approximate radiation dose that astronauts may be exposed to with long‐term space travel. Unfortunately, this study was limited by its low sample size of four rats per treatment groups. However, there is still a trend demonstrating that low dose radiation may have a deleterious effect on bone strength that can be further assessed in the future. As previously mentioned, this study used rats from a pilot study done by another investigator who was evaluating blood glucose status in the rats; this constrained the number of rats available for this study.

This study used the same X‐ray cabinet as Chowdhury et al., (2016), who previously found that a total cumulative dose of 6 GY over 2 weeks did not further attenuate (beyond hindlimb suspension) rat femur and tibia bone strength (i.e. three‐point bending) or bone mineral density. However, in our study rats were suspended for 4 weeks after being exposed to a single dose of 1 Gy. Overall, our biophysical parameters suggest low dose radiation can have a deleterious effect on the tibia, but not the femur. This is not surprising considering the tibia is anatomically slender and has a thinner cortex than the femur (Table 1). Also, the osteoclast and osteoblast activity involved in bone remodelling occurs along bone stress lines (which is different between the tibia and femur) to support body weight and force of movement (Bergstrom et al., 2017; Hart et al., 2020). Interestingly, Bandstra et al., (2008) exposed mice (not suspended) to a single dose of 0.5, 1 or 2 GY whole‐body radiation. After 16 weeks they started to find detectable cortical tibial bone loss at 1 GY exposure using microcomputed tomography; there was no effect on the biomechanical properties of stiffness and flexure force. Similar to our study, noticeable effects were confined to the tibia and not the femur. Likewise, Zhang et al., (2018), exposed rats to incremental whole‐body radiation for a cumulative dose of 4 GY. After 4 weeks, they reported no significant effect of radiation on the femur flexure force, Young's modulus/elasticity, and bone mineral density (they did not evaluate the tibia).

Young's modulus (elasticity) offers a better tissue‐level assessment of bone strength because it measures the resistance to deformation (Jepsen et al., 2015). Calculation of this modulus integrates in bone stiffness and the moment of inertia, in which the moment of inertia represents the geometric anatomy of the bone along an axis at the point of bending/loading. As mentioned in the Results section, the tibia geometry is a limitation in calculating the elastic modulus. In contrast, stiffness just evaluates how much the bone diaphysis deforms with pressure/load. This study only evaluated bone at its mid‐diaphysis, but bone is quite heterogeneous along its length (Bergstrom et al., 2017). Bone remodelling due to a compressive load is different between the cranial and caudal sides (Bergstrom et al., 2017). Also, Gamboa et al., (2021) used rats exposed to spaceflight and found that within the same long bone there were differences between the head, metaphysis and epiphysis during the process of osteopenia. Using a single dose of 2 GY whole body radiation exposure, Hamilton et al., (2006) found no impact of radiation on cortical porosity and thickness of the mouse femur 110 days after radiation exposure; the degeneration appeared to happen only at the trabeculae of the bone epiphysis.

Overall, our study of 1 GY whole body, X‐ray radiation had a negative impact on the biophysical features of the tibia. Although the mineral composition does not appear to be affected, we assume that radiation likely induced some structural changes for the effects demonstrated; future studies will be needed to fully understand what changes are induced with radiation. We suspect that a greater dose of radiation would have induced a more deleterious effect on the flexure force, stiffness and elasticity of both bones. Interestingly, Bandara et al., (2008) suggested that the bone deterioration threshold in mice is between 0.5 and 1 GY. Therefore, this study is a good first step, but more advanced methods will need to be used at the micro‐scale to determine how bones respond differently to radiation.

## AUTHOR CONTRIBUTIONS

Rahul Mehta and Brent J. F. Hill were responsible for the experimental study done at the University of Central Arkansas. Sample preparation, data collection and analysis were performed by both authors. The manuscript was co‐written by Rahul Mehta and Brent J. F. Hill. Both authors have read and approved the final version of this manuscript and agree to be accountable for all aspects of the work in ensuring that questions related to the accuracy or integrity of any part of the work are appropriately investigated and resolved. All persons designated as authors qualify for authorship, and all those who qualify for authorship are listed.

## CONFLICT OF INTEREST

None declared.

## GENERATIVE AI STATEMENT

No AI tool or application was used to generate this manuscript.

## Data Availability

Any datasets or raw data not included in the body of this manuscript is available from the corresponding author based upon reasonable request.
